# The Landscape of Actionable Gene Fusions in Colorectal Cancer

**DOI:** 10.3390/ijms20215319

**Published:** 2019-10-25

**Authors:** Filippo Pagani, Giovanni Randon, Vincenzo Guarini, Alessandra Raimondi, Michele Prisciandaro, Riccardo Lobefaro, Maria Di Bartolomeo, Gabriella Sozzi, Filippo de Braud, Patrizia Gasparini, Filippo Pietrantonio

**Affiliations:** 1 Medical Oncology Department, Fondazione IRCCS Istituto Nazionale dei Tumori, 20133 Milano, Italy; filippo.pagani@istitutotumori.mi.it (F.P.); giovanni.randon@istitutotumori.mi.it (G.R.); vincenzo.guarini@istitutotumori.mi.it (V.G.); alessandra.raimondi@istitutotumori.mi.it (A.R.); michele.prisciandaro@istitutotumori.mi.it (M.P.); riccardo.lobefaro@istitutotumori.mi.it (R.L.); maria.dibartolomeo@istitutotumori.mi.it (M.D.B.); filippo.debraud@istitutotumori.mi.it (F.d.B.); 2 Unit of Molecular Cytogenetics, Fondazione IRCCS Istituto Nazionale Tumori, 20133 Milano, Italy; gabriella.sozzi@istitutotumori.mi.it (G.S.); patrizia.gasparini@istitutotumori.mi.it (P.G.); 3 Department of Oncology and Hemato-Oncology, University of Milan, 20122 Milan, Italy

**Keywords:** gene fusions, colorectal cancer, translocation, biomarker

## Abstract

The treatment scenario of metastatic colorectal cancer (mCRC) has been rapidly enriched with new chemotherapy combinations and biological agents that lead to a remarkable improvement in patients’ outcome. Kinase gene fusions account for less than 1% of mCRC overall but are enriched in patients with high microsatellite instability, *RAS/BRAF* wild-type colorectal cancer. mCRC patients harboring such alterations show a poor prognosis with standard treatments that could be reversed by adopting novel therapeutic strategies. Moving forward to a positive selection of mCRC patients suitable for targeted therapy in the era of personalized medicine, actionable gene fusions, although rare, represent a peculiar opportunity to disrupt a tumor alteration to achieve therapeutic goal. Here we summarize the current knowledge on potentially actionable gene fusions in colorectal cancer available from retrospective experiences and promising preliminary results of new basket trials.

## 1. Introduction

Colorectal cancer represents the third most common tumor in men and the second in women, with an estimated incidence of approximately 1.8 million new cases and a total number of about 860,000 deaths worldwide in 2018 [1]. The treatment landscape of mCRC has undergone a dramatic change in the last decades, allowing to reach a remarkable improvement in patients’ prognosis, thanks to the introduction of biological agents (anti-angiogenic and anti-Epidermal Growth Factor Receptor (EGFR)) [2] and the development of continuum of care strategies across several available treatment options [3,4]. Nowadays, the decision-making process on the optimal treatment approach in mCRC patients should carefully consider both the patients’ characteristics and the tumor’s molecular profile [5]. Specifically, all the major guidelines recommend the testing of the mutational status of *RAS* and *BRAF* genes [6,7] since *RAS* mutation is a validated negative predictive marker for anti-EGFRs response, and *BRAF V600E* mutations are associated with a very limited benefit from EGFR inhibitors [8].

Nevertheless, the recent progress in tumor genomic profiling has shed light on the complex molecular scenario of mCRC [9], identifying a wide range of genomic alterations (mutations, translocations, or amplifications) that could play both a prognostic role, conferring a higher aggressiveness to the tumor, and a predictive one, identifying tumors with primary refractoriness to biologic agents, with the aim to better select patients for the different treatment options [10,11]. Moving forward, the new molecular aberrations identified may constitute a driver for tumor initiation and progression, thus potentially representing new therapeutic targets, allowing to reach a deeper treatment personalization. 

In this setting, the emerging role of genomic translocations has to be emphasized. Gene fusions represent an important piece of the puzzle of the tumor genomic landscape and are involved in the development of about 16% of all cancer types [12]. In fact, in some cases, they display a close correlation with a specific tumor subtype, thus representing a diagnostic marker (e.g., *FLI1/EWS* in Ewing Sarcoma), while in others they are endowed with a prognostic value, providing a risk stratification (e.g., the presence of gene fusions in embryonal rhabdomyosarcoma), or they could represent a potential therapeutic target (e.g., *ALK* and *ROS1* in non-small cell lung cancer (NSCLC)) [13]. In the last decades, an increasing number of gene rearrangements has been identified in several cancer types, including CRC, thanks to new techniques of high-throughput genome sequencing, although the available body of knowledge on gene fusions in tumors is still incomplete [14]. Specifically, the key challenge is to distinguish the genomic aberrations that represent true oncogenic drivers from the passenger alterations that do not play a role in the cancer development and progression. Potential distinction factors could be the biological function of the genes involved and the type of rearrangement (juxtaposition to the promoter of a highly-expressed gene or the presence of a continuous open reading frame with functional domains, such as kinases) [15]. 

However, the low frequency of the singular novel genomic alterations and gene fusions is a limiting factor for their thorough study from a pathogenic and therapeutic perspective, hampering the investigation in dedicated clinical trials and the translation in the clinical practice setting [16]. In this light, the paradigm of cancer research is undergoing a deep change: from a tumor type-focused approach to a molecularly-directed agnostic one, exploring the role of biologic agents targeted to the driver genomic alteration irrespectively of the cancer histology [12]. Thanks to the recently-conducted basket trials, clinical studies in which patients affected by several tumor types harboring the same genomic aberration received a specific targeted treatment, the tyrosine kinase inhibitor (TKI) larotrectinib was granted accelerated approval by Food and Drug Administration (FDA) for cancers displaying *NTRK* pathogenic fusions while entrectinib, a TKI targeting *NTRK*, *ALK* and *ROS1* fusions, was granted priority review by FDA [17,18].

Besides this tissue-agnostic approach, the deep understanding of the genomic profile of the singular tumor types is still crucial in order to identify subgroups of patients with an enrichment of peculiar molecular drivers and to better select the optimal candidates for genomic testing and targeted treatment. Specifically, in CRC, a recent comprehensive analysis of 2.314 cases showed how the frequency of kinase gene fusions in an unselected population is about 0.9%, but it significantly increases in MSI-high (MSI-H) tumors (5%) and overall in *RAS/BRAF* wild type cancers (15%) [19]. Preclinical data and translational studies have shown the potential importance of the identification of patients affected by tumors harboring molecularly altered kinase genes, since they represent a population unlikely to respond to anti-EGFR treatment but may benefit from selective targeted agents [20,21]. However, further studies are warranted to validate this evidence through prospective, ad-hoc-designed clinical trials and to potentially change the treatment paradigm in these selected populations.

In this review we aim at summarizing the available literature evidence on the genomic fusions in CRC, with a specific focus on those harboring a prognostic and/or predictive value or especially representing potentially druggable targets in the metastatic disease setting.

### 1.1. NTRK

Neurotrophic receptor tyrosin kinase genes *NTRK-1*, *NTRK-2*, *NTRK-3* (encoding for TRK-A, TRK-B, and TRK-C respectively) are involved in neuronal tissue homeostasis and expression is mainly confined to the nervous system in adult tissues. Ligands include neurotrophins such as NGF (for TRK-A), BDNF and NF4 (for TRK-B) and NT3 (for TRK-C) [22]. Fusions account for the most common oncogenic activation of *NTRK* genes and are found at a low frequency in colorectal cancer (i.e., around 0.9%) [23]. 

The presence of *NTRK* fusions may be shown by extended next-generation sequencing, targeted RNA sequencing, and fluorescent in situ hybridization, while immunohistochemistry (IHC) is performed as a screening method to detect the overexpression of any TRK receptor (A, B, or C), a result that reflects the gene fusion. Whilst IHC may represent a rapid and costless screen for *NTRK* fusions endowed with high specificity [24,25], caution should be taken for MSI-H CRC tumors as mutations within the epitope recognized by pan-TRK antibody might impair specificity of the assay [26]. 

In CRC, *NTRK* fusions (Table 1a) are associated with female gender, elderly age, right sidedness, lymph node metastatic spread, MSI-H, and *RAS* and *BRAF* wild-type status as well as dismal prognosis with median overall survival (OS) of around 15 months in the metastatic setting [27]. Beyond bearing a well-defined prognostic role *NTRK* fusions are likely to account for primary resistance to EGFR targeted agents [21] due to downstream mitogen-activated kinases (MAPKs) activation [28].

Several NTRK-targeted TKIs are at different stages of clinical development, or TKI with anti-TRK activity are available whose labeling is limited to different indications than treatment of *NTRK* fusion positive tumors (Table 2) [22]. The oral TRK-selective inhibitor larotrectinib was granted tissue-agnostic FDA approval on November 2018 based on results of single-arm trials LOXO-TRK-14001, SCOUT and NAVIGATE [18]. Among 55 treated patients, including 4 patients with metastatic CRC (mCRC), objective response rate was as high as 75%; in the latter group RECIST partial response and disease control rate were achieved in 2 and 4 cases, respectively. Clinical benefit was seen regardless of specific *NTRK* fusion partner. Entrectinib is an oral inhibitor of NTRK, ALK, and ROS1 [38]. Entrectinib has been tested in the single-arm trials ALKA-372-001, STARTRK-1 [39], STARTRK-02 [40], and STARTRK-NG [41]. Among treated patients with *NTRK*-fusion positive tumors, 1 was diagnosed with a *LMNA-NTRK1* rearranged mCRC and achieved a 12-week lasting partial response.

Collectively these data suggest that extended screening in selected mCRC patients should be recommended as targeted therapies might improve the otherwise poor prognosis of patients with tumors bearing *NTRK*-fusions.

### 1.2. ALK and ROS1

Anaplastic lymphoma kinase (*ALK*) and v-ros avian UR2 sarcoma virus oncogene homolog (*ROS1*) encode tyrosine kinases that are constitutive activated by fusion events leading to cellular transformation and promoting survival and proliferation through downstream signaling. Nearly 30 different ALK fusion protein partners have been described, with echinoderm microtubule-associated protein-like 4 (EML4) and nucleophosmin (NPM) being the most prevalent in NSCLC and anaplastic large cell lymphoma (ALCL) occurring in 3% to 5% and more than 50%, respectively [58]. *CD74-ROS1* fusion gene have been reported as *ROS1* most common rearrangement in NSCLC for approximately 1% [59]. Although ROS1 and ALK tyrosine kinase domains share significant homology and their genes rearrangements are mutually exclusive, both have similar clinicopathological characteristics with high prevalence in younger, non-smoking patients with adenocarcinoma histotype [58,59,60].

*ALK* and *ROS1* rearrangements (enlisted in Table 1a) rarely have been also identified in mCRC using technologies based on fluorescence in situ hybridization (FISH) [61], exon array profiling [62], and next generation sequencing (NGS) [14,33] performed on archival tumor specimens.

Two studies evaluated the presence of the classic *ALK-EML4* fusion gene by FISH, in the absence of any enrichment strategy to improve the detection rate, without evidence of rearrangement in metastatic colorectal cancer patients (0 of 770 [63], 0 of 17 [33]). Other investigators that utilized immunohistochemistry (IHC) as screening strategy to identify *ALK* rearrangements, found 0.44% to 2.4% IHC and subsequent FISH positive cases [20,34,64,65]. Additionally, new gene fusions who may be potentially responsive to target therapy were identified through NGS. Lipson et al. described one case of *C2orf44-ALK* fusion mCRC [32]; moreover, Ying et al. reported *SPTBN1*, a gene encoding for a cytoskeletal protein, as a potential *ALK* fusion partner [66]. Recently we retrospectively collected 14 cases of CRC harboring *ALK* (11 cases) and *ROS1* (3 cases) fusions, along with 13 CRC cases harboring *NTRK* fusion, comparing with a cohort of 319 patients not bearing rearrangements [27]. The main finding of our work was the identification of clinical features and molecular characteristics of patients with these rearranged tumors already listed in the previous section. Further two rearrangement-positive cases of *ROS1* were also described by Aisner et al. (2/236), suggesting that *ROS1* gene may also be activated by gene fusion in CRC [67].

To date, crizotinib, a small molecule ATP analogue inhibitor of ALK, ROS1, and MET, is approved by FDA, EMA, and AIFA for the treatment of patients with ALK or ROS1-positive NSCLC [42,47]. Further examples of ALK TKIs include alectinib, ceritinib, brigatinib, and lorlatinib; all binding the ATP-pocket of the ALK kinase domain differently, showing various profiles of inhibition [58]. Alectinib was approved by FDA, EMA, and AIFA as first line treatment for ALK positive NSCLC based on results of randomized phase III ALEX study comparing alectinib vs crizotinib [43]. Moreover, third generation inhibitor of ALK and ROS1 tyrosine kinases lorlatinib was approved for second- or third-line treatment of ALK-positive metastatic NSCLC [46]. Patients with solid tumors harboring ROS1, ALK, or NTRK1/2/3 molecular alterations could also benefit from treatment with entrectinib [39] (Table 2).

*ALK* and *ROS1* rearrangements are new molecular subgroup of poor prognosis mCRC whose recognition allows a proper tailored management. New targeted strategies may be a promising treatment to be further investigated.

### 1.3. RET

The rearranged during transfection (*RET*) gene, encodes for a single-pass transmembrane receptor tyrosine kinase (RTK) required for the development of neural and genitourinary tissues. Its signaling is mediated by the bound of soluble proteins of the glial cell line-derived neurotrophic factor (GDNF) family ligands (GFLs), such as neurturin, artemin, and persephin. RET is not able to bind GFLs, but it needs an additional co-receptor that is the GDNF family receptor-α (GFRα). This complex mediates RET homodimerization, trans-autophosphorylation of tyrosine residues within the RET intracellular domains, and the subsequent activation of signal transduction cascades including the MAPK, PI3K, JAK–STAT, PKA, and PKC pathways [68].

Oncogenic activation of *RET*, causing heritable and sporadic cancers, results of either activating point mutations or genomic rearrangements with the production of chimeric RET proteins that lead to its constitutive activation or through aberrant expression or activation of wild-type receptors. Rearrangements mostly involve the long arm of chromosome 10 with inversions or translocations of *RET* 3′ kinase domain encoding sequences, with the 5′- ones from several partner genes which encode protein dimerization domains. To date, more than 13 different *RET* translocations have been identified; however, those linking *RET* with the coiled-coil domain-containing 6 (*CCDC6*), nuclear receptor co-activator 4 (*NCOA4*), or with kinesin family member 5B (*KIF5B*) are the most reported [68,69]. *CCDC6–RET* and *NCOA4–RET* are the most commonly identified *RET* fusions in papillary thyroid cancers where *RET* rearrangements seem to be correlated with a more aggressive phenotype [70]. Otherwise, *KIF5B– RET* is the most commonly identified *RET* fusion in NSCLC associated with younger, never-smoker patients and poorly differentiated tumors [32,71,72].

*RET* fusions, although rare, are characteristic of a small subset of mCRC patients. Retrospective case series showed that *RET* rearrangements occurs in about the 0.2% of mCRC patients, and they are mutually exclusive with other driver mutations [73]. In a recently small series of 24 *RET*-rearranged mCRC patients, we observed that *RET* fusions were mainly *NCOA4-RET* and *CCDC6-RET*, while describing for the first time *TNIP1-RET* and *SNRNP70-RET* fusions. Moreover, we showed that *RET* fusions were more frequent in older patients, with Eastern Cooperative Oncology Group performance status (ECOG PS) 1–2, right-sided, unresected primary tumors, and they were associated with a significantly shorter OS when compared with 291 *RET*-negative patients (median OS 14.0 versus 38.0 months, HR: 4.59; 95% CI, 3.64–32.66; *p* < 0.001) [74]. Known RET partner genes are summarized in Table 1a.

Several multikinase inhibitor drugs showed activity against RET in preclinical and clinical studies [75]. Some of them have been approved by the FDA for the treatment of cancer (Table 2). In particular, cabozantinib [76], vandetanib [48], lenvatinib [49], and sorafenib [77] gained the approval for the treatment of medullary thyroid carcinomas and differentiated thyroid cancers while alectinib [43] has been approved in lung adenocarcinomas and regorafenib [53] in CRC. None of these drugs was designed to bind preferentially RET; for this, RET-specific antagonists are awaited to enrich the treatment armamentarium, to perform better clinical outcomes and to limit the consequences of the off-target toxicities caused by a multikinase inhibition, such as the adverse events (AEs) related to antiangiogenic activity [75]. Two small molecules with selective anti-RET activity, BLU-667 [78] and LOXO-292 [79], showed impressive results in preclinical models and are currently being evaluated in two ongoing basket trials, ARROW [80] and LIBRETTO-001 [81], respectively, that enroll patients with advanced, RET-altered solid tumors, that have progressed or are intolerant to available standard therapies. Preliminary results from 35 patients with *RET*-fusion positive tumors (27 NSCLC, 7 papillary thyroid cancer, 1 pancreatic cancer), treated with LOXO-292 showed a good tolerability, with none AEs graded ≥3, and an overall response rate (ORR) of 69%. NSCLC responses occurred independent of upstream partner when known [56]. Similar preliminary results have been achieved with BLU-667 in *RET*-fusion positive NSCLC [54] and thyroid cancer patients [55]. With respect to the mCRC patients’ cohorts, unfortunately no data from clinical trials are currently available, but a case report of a patient with mCRC harboring a *RET* fusion treated with a selective RET inhibitor has been reported. The patient affected by a right sided, MSI-high, and *CCDC6-RET* positive mCRC achieved a complete response to the selective *RET* inhibitor drug RXDX-105 with an impressive PFS of 19 months [74]. Hope and confidence are rapidly growing around these new therapeutic options that could guarantee a great clinical benefit, although restricted to a small subset of patients.

### 1.4. BRAF

*BRAF* gene encodes for a protein belonging to the RAF family of serine/threonine protein kinases. This protein plays a role in regulating the MAP kinase/ERK signaling pathway, which affects cell division, differentiation, and secretion. Activating oncogenic mutations in this gene, above all V600E, are encountered in a wide range of neoplasms and are mutually exclusive with mutations in *KRAS/NRAS* genes in mCRC [82]. Patients with BRAF V600E mutated mCRC derive limited benefit from standard therapies, have poor prognosis, and share peculiar clinical and pathological characteristics: they are frequently women with right-sided primary and mucinous histology [83]. Moreover, the presence of BRAF V600E mutation is frequently associated with a microsatellite instability status and spread to lymph nodes and peritoneum, characteristics that overlap those of mCRC enriched in gene fusions [19,27,31,74].

Activating *BRAF* gene fusions are the most prevalent genetic alteration in pediatric pilocytic astrocytoma [84] and are commonly detected in pediatric papillary thyroid carcinoma [85]. Acquisition of a *BRAF* fusion has recently been described as a novel mechanism of acquired resistance to vemurafenib in a patient with BRAF V600E melanoma, underlining the key role of this oncogenic activation in cancer survival [86]. Furthermore, *BRAF* gene fusions have been detected in around 4% of skin melanoma without recurrent driver mutations in oncogenes, with preliminary evidences of response from MEK inhibitions [87].

Concrete efforts have been carried out in order to describe *BRAF* gene fusions in colorectal cancer (Table 1a). Ross et al. [88] analyzed 20,573 tumor samples, across 12 different kinds of solid tumors, and among them 2154 colorectal cancers, 4 of which (0.2%) bearing *BRAF* translocations (1 *AGAP3-BRAF* and 1 *MKRN1-BRAF* in primary tumors; 2 *TRIM24-BRAF*, both in metastatic lesions). Both female and male patients were involved (53% and 47% respectively), with a median age of 56 years [88]. Kloosterman et al. found among 278 primary colon cancer samples 3 different *BRAF* gene fusions: *AGAP3-BRAF* and *TRIM24-BRAF* (caused by an inversion event) and *DLG1-BRAF* (deriving from a reciprocal translocation between chromosomes 3 and 7), all of them identified in wild-type *BRAF/KRAS/NRAS* patients [16].

Unfortunately, no data are currently available on targeted therapy (e.g., with a MEK inhibitor agent) for patients with *BRAF* rearranged mCRC, underlining the urgent need to produce evidences supporting this novel therapeutic paradigm.

### 1.5. Other Gene Fusions: An Emerging Complexity

There are some subsets of colorectal tumors that express other gene fusions, some of which, however, are extremely rare or not yet therapeutically actionable (Table 1b).

Mutations and fusions in the gene encoding for the fibroblast growth factor receptor (*FGFR2/3)* are common in patients with urothelial carcinoma, accounting for around 20% in the advanced setting of the disease [89]. The treatment of patients affected by this tumor has been recently enriched by the small molecule erdafitinib, that received the FDA accelerated approval based on the results of a phase 2 study [57]. In mCRC, *FGFR* fusion are rare and occasionally reported (Table 1a) [19], but again, patients with mCRC harboring this specific alteration could benefit from a targeted treatment already available.

Selvam and colleagues evaluated gene fusions in 183 solid tumor samples by means of RNA sequencing [35]. In the CRC cohort of 18 cases, they identified two novel fusion gene transcripts, SARAF (TMEM66)-NRG1 and FGFR3-SPDYE4. In the same cohort the authors described two R-spondin fusions, EIF3E-RSPO2 and PTPRK-RSPO3, involving the R-spondin family members RSPO2 and RSPO3, already described by Seshagiri et al. [90] An ongoing phase I trial (NCT01351103) [91] is evaluating the dose escalation of oral LGK974 in patients with malignancies dependent on Wnt ligands. Another RNA sequencing study evaluating 147 CRC samples described 24 fusion gene transcripts, including novel fusions, 13 of which involved oncogenic fusions (e.g., GTF3A-CDK8, NAGLU-IKZF3, RNF121-FOLR2) whose overexpression correlates with cell proliferation [36]. Jang JE and colleagues performed RNA sequencing on 28 CRC cell lines describing a novel gene fusion transcript (NFATC3-PLA2G15) in two of them and demonstrating its contribution to tumor progression [37].

Human epidermal growth factor receptor 2 (HER2) is a member of the epidermal growth factor receptor (EGFR) family that activates subsequently to the heterodimerization with other ligand-bound receptors, such as HER3, triggering multiple downstream pathways, among which MAPK, PI3K-Akt are the main ones [92]. HER2 rearrangements are rare and reports are anecdotic. Ross et al. identified two patients one with lobular breast cancer and the other with urothelial bladder carcinoma with the *ERBB2-GRB7* fusion subsequent to a deletion of an 18-kb segment on chromosome 17 [93,94]. The same fusion was also described by Chmielecki et al. in a bladder cancer and a breast cancer patient [95]. In all cases this rearrangement was mutually exclusive with other *ERBB2* alterations. To our knowledge, only a HER2-rearranged mCRC sample as result of the same *ERBB2-GRB7* fusion has been reported in the literature. A possible explanation of this lack of detection is that IHC testing for the fusion protein produces negative results due to the deletion of the IHC epitope in the altered protein, compromising a low-cost screening by this test. Unfortunately, the HER2-rearranged mCRC patient did not achieve a response from anti-HER2 therapies, but further evidences are needed to assess the functional and therapeutically significance of *ERBB2* fusions [30]. Several HER2 inhibitors, such as trastuzumab, pertuzumab, and lapatinib, are routinely employed in the treatment of *HER2* amplified breast and gastroesophageal cancers, and they might be active even in solid tumors with *HER2* activating mutations [96]. Recently, an increasing consideration has been given to the oncogenic *HER2* activation in CRC where its over-expression has been reported in 2% considering CRC overall, increasing up to 5% in stage III and IV KRAS exon 2 wild-type tumors [97,98,99]. *HER2* gene amplification and *HER2* activating mutations have been implicated as mechanisms for cancer cell resistance to anti-EGFR therapies in mCRC and, even if it is still debated, as negative prognostic biomarkers [100]. Furthermore, underlining the relevance of this oncogene activation in mCRC, the recent HERACLES and MYPATHWAY trials have established the clinical benefit in *HER2* amplified mCRC patients treated with HER2 inhibitors [101,102]. *HER2* fusions might be a potential target of inhibition; however, to date, there are not published or ongoing trials with HER2 inhibitors in patients with *HER2*-rearranged tumors.

### 1.6. Methods to Detect Gene Fusions

Fusion events, involving a variety of partner genes, result in the generation of chimeric fusion kinases with oncogenic transformation potential and induction of oncogene dependency within the neoplastic cells [103]. For all these reasons, their rapid and accurate detection can stratify cancer diagnosis and inform clinical action, while predicting prognosis, patient survival and treatment response [104].

Currently, conventional methods to detect clinically ‘actionable’ fusion genes in routine diagnostics rely on fluorescent in situ hybridization (FISH), immunohistochemistry (IHC), and/or quantitative real-time polymerase chain reaction (RT-PCR). While FISH, and at times IHC, is highly sensitive and considered the gold standard for several gene-testing (i.e., ALK), all these methods are low-throughput and typically only test for the presence of a single fusion gene, often resulting in a lengthy, tissue consuming, repetitive, and costly path to diagnosis [104,105]. As a consequence, only the recurrent fusion genes are iteratively tested during diagnosis [104]. Moreover, analysis and scoring systems of FISH signals as well as staining intensities of IHC require several skills and standard operating procedures in order to assure high-level quality of test results. These methods also rely on experienced and trained professionals, as FISH and IHC are challenged by subjective interpretations of results [104,105]. Another limitation to be considered is that neither FISH nor IHC provide information of the exact molecular breakpoints and, most of the times, cannot detect their partner genes. Indeed, defining the fusion partner gene is critical to refine treatment strategy, unveil the biology behind the genetic rearrangements and, most importantly, provide information on therapy response [105]. On the other hand, RT-PCR can overcome some of the issues presented by FISH and IHC, as it is a very sensitive method and the only one able to precisely detect fusions, including identification of both partner genes and the exons involved. However, RT-PCR is mainly focused on only the most frequent fusion events and cannot identify rare exon combinations. The great disadvantage of this methodology remains the limited multiplexing capability [103,106].

In contrast to the traditional techniques, high-throughput transcriptome analysis and next generation sequencing (NGS) can deliver high-resolution fusion gene detection while assessing hundreds of genes in a single test, identifying both known and novel fusion genes (including rare fusions and novel gene partners) [103,104,105,107,108]. Several studies have provided evidence that these techniques not only provide the same sensitivity and specificity as conventional diagnostic methods but also rely on multiplexing potential, allowing to screen larger selection of actionable targets, with small amount of tissue sample [19,103,108]. The diagnostic power and the ability of targeted RNA-seq to identify different fusion genes as compared to FISH and RT-PCR methods, was demonstrated by Heyer et al. in a clinical cohort comprised of hematological and solid tumors (prostate, sarcoma, and lung cancers). Although RNAseq can overcome many limitations of the traditional techniques, it still suffers from poor sensitivity for detecting fusion genes that are lowly expressed or diluted by accompanying non-cancerous cells within a sample, due to the total size of the transcriptome [104]. Plenty of works demonstrate the utility of different trascriptome-based platforms (i.e., Nanostring Elements, Agena LungFusion panel, ThermoFisher NGS fusion panel, Archer NGS, Ion Torrent AmpliseqTM Colon, and lung cancer panel) commercially available for the identification of rearrangements in a large set of genes, all revealing a good concordance of results compared with FISH analysis [19,103,108]. Overall, an NGS approach can offer many advantages for diagnostic laboratory testing several cancers samples: (a) allows detection of multiple fusions in a single assay and can easily also be multiplexed with detection of point mutations and small insertions and deletions; (b) the single-assay format allows for faster turn-around-time and lower cost than doing the assays separately; (c) utilization of small amount of input RNA required is very valuable for these samples [19,103,104,108].

**Figure 1:**
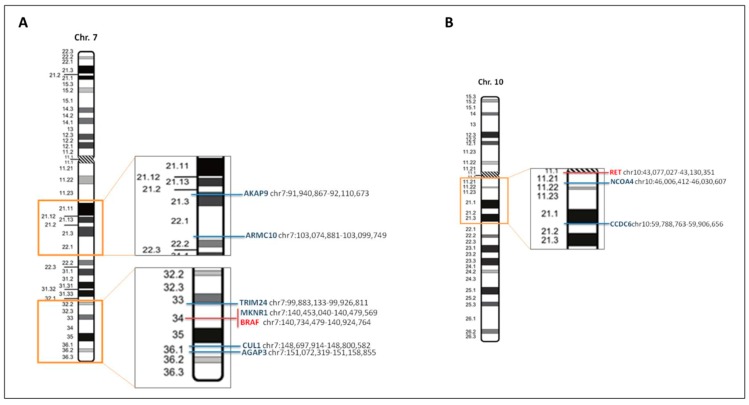
Genetic map of BRAF and RET and their partner genes. (**A**) Genetic and physical map of the chromosome 7, indicating (in the enlarged rectangles) the location of BRAF (in red) and some of its gene partners (in blue). (**B**) Moreover, for chromosome 10, containing RET (in red), some of the intrachromosomal genes partners are indicated in blue. Maps and gene location are derived from the website of the University of California Santa Cruz Genome Browser (http://genome.ucsc.edu/), with adaptations.

In colorectal cancer, several fusion genes are generated by small intrachromosomal inversions, deletions or insertions making split-apart FISH signals very challenging, if not impossible, to visualize. For instance, as illustrated by Figure 1A, complex intrachromosomal fusion rearrangements involving *BRAF* and some of its partner genes, in particular *MKNR1* and *TRIM24* present difficult interpretation of FISH analysis considering that the distance between them is quite small. A similar scenario is presented by gene fusions created with *RET* (Figure 1B) where *NCOA4* and *CCDC6* are also located close to each other, and *RET* and split-signals indicating presence of rearrangement could be easily missed. Considering these limitations, in addition to all the previously mentioned ones, ‘actionable’ fusion genes in colon cancer should be investigated utilizing high-throughput methods such as targeted NGS platforms, either specifically customized for colon cancer or utilizing other panels containing most of the targets of interest.

Lastly, as novel fusion genes or fusion partners are detected, and their clinical implication is confirmed, a periodical update of the primer pool would facilitate the continuing utility of this assay, inclusive of all potentially targetable gene fusions in cancer.

## 2. Discussion

We focused on oncogenes known to drive carcinogenesis, aggressiveness of tumor, and whose activating alterations such point mutations or gene amplifications are in some cases already targetable in mCRC. As described, fusions and mutations are mutually exclusive, both leading to an over-activation of the gene involved, and either of these aberrations may render the tumors sensitive to therapeutic targeting.

To date, the therapeutic algorithm for the choice of the best treatment for mCRC patients has been characterized by a negative selection, restricting the treatment with anti-EGFR agents to patients with wild-type *RAS* and *BRAF* mCRC [109,110]. Moving forward the era of positive selection in mCRC, results from the phase III BEACON trial, which randomized 665 pretreated patients with *BRAF* V600E mutant mCRC to receive triplet therapy (encorafenib, binimetinib and cetuximab), doublet therapy (encorafenib and cetuximab), or the investigator’s choice of irinotecan or FOLFIRI and cetuximab, showed an impressive median overall survival of 9 months (95% confidence interval (CI): 8, 11.4) for the triplet targeted therapy compared to 5.4 months (95% CI: 4.8, 6.6) for standard therapy (hazard ratio (HR) 0.52; 95% CI: 0.39, 0.7, *p* < 0.0001) and an ORR of 26% (95% CI: 18, 35) for the triplet targeted therapy compared to 2% (95% CI: 0,7, *p* < 0.0001) for standard therapy [111,112]. An analogous approach, with the inhibition of the entire axis of EGFR signal transduction, could be effective in mCRC patients harboring a *BRAF* gene fusion.

Focusing on molecular biomarkers leading to a positive selection of mCRC patients, the phase II HERACLES study showed the activity of the combination of trastuzumab and lapatinib in HER2-amplified heavily pretreated mCRC patients [101], demonstrating that targeting HER2 is a successful therapeutic strategy in mCRC, to be tested in HER2-rearranged mCRC patients.

However, gene fusions detected with deep sequencing may represent chance events caused by tumor’s chromosomal instability, with poor pathogenic and druggable significant, underlining the need to classify gene fusions by functional assays. The poor cost-effectiveness of assessing such rare alterations in the daily clinical practice represents another limitation to the tailored treatment of mCRC patients harboring gene fusions. Nevertheless, selection criteria for screening of patients who may bear a gene fusion are already available thanks to retrospective efforts that identified clinical and pathological characteristics linked to the presence of such alterations. Furthermore, the importance of an early recognition of specific gene rearrangements is underlined by their negative prognostic role and potential negative predictive impact from anti-EGFR treatment, characteristics possibly driven by the high presence of microsatellite instability status in this patient population [19,21,27,74].

Given these already available evidences, it seems reasonable to screen patients with a new diagnosis of *RAS/BRAF* wild-type mCRC for gene fusions by means of IHC testing and hopefully in the next future by targeted NGS panels. The importance of an early detection of potentially druggable fusions is underlined by the unfavorable prognostic role of such alterations. In fact, the reduced life expectancy of affected patients may significantly impair their chances to receive a targeted treatment and to be enrolled in dedicated clinical trials. Less evidences are available to determine the best first-line standard treatment for mCRC patients harboring a kinase gene fusion. Nevertheless, the aggressive clinical course of these tumors and the limited benefit achieved with anti-EGFR treatments in patients with *ALK-ROS1-NTRK-* or *RET-* rearranged mCRC [21,113] may suggest an intensified chemotherapy regimen such the triplet FOLFOXIRI plus bevacizumab as the best first-line treatment, while the enrollment in a fusion-targeted clinical trial should be considered as long as available.

As previously underlined, *BRAF/RAS* wild-type, MSI-H mCRC are enriched for targetable gene fusions [19,31]. In detail, only MSI-H mCRC in which *MLH1* expression has been silenced by the gene promoter methylation are associated with an enrichment of kinase fusions, rather than MSI-H tumors caused by germline or somatic mutations in MMR genes [31]. Therefore, these data suggest that the prevalence of Lynch syndrome in MSI-H tumors harboring gene fusions may be extremely low, mirroring what has been demonstrated for BRAF V600E mutations. Immune-checkpoint inhibition either with pembrolizumab or nivolumab monotherapy and with nivolumab plus ipilimumab combination has been recently approved by FDA for the treatment of MSI-H mCRC patients, with response rates ranging from 31% to 60% [114,115,116,117]. No data are currently available on the differential outcomes between MSI-H mCRC patients with or without gene fusions, even if responses to treatment are usually independent from the presence of other driver alterations such as *KRAS* or *BRAF* mutations. However, the combination of targeted agents and immunotherapy could be a successful strategy [118] and deserves further investigations in MSI-H, rearranged mCRC patients, even if the extremely low prevalence of such molecular subgroups challenges the real-world feasibility of dedicated clinical trials.

In conclusion, actionable gene fusions are rare in mCRC overall but reach a significant percentage in a selected population of patients that could benefit from an extremely active treatment either with a targeted agent or in combination with immune-checkpoint inhibitors.

## Figures and Tables

**Table:** (**a**)

**Fusion Partners**	**NTRK1-**	**NTRK2-**	**NTRK3-**	**ALK-**	**ROS1-**	**RET1-**	**BRAF-**	**FGFR2-**	**FGFR3-**	**ERBB2-**
	LMNA [29]	DAB2IP [29]	ETV6 [29]	SPTBN1 [19]	GOPC [27]	CCDC6 [27]	AGAP3 [19]	MYH15 [19]	STAB1 [19]	GRB7 [30]
	TPM3 [29]		EML4 [16]	CAD [27]	SLC34A2 [27]	GEMIN5 [19]	TRIM24 [19]		SPDYE4 [19]	
	SCYL3 [29]		KANK1 [31]	EML4 [27]		NCOA4 [19]	CUL1 [19]			
	PLEKHA6 [29]			CENPF [27]		RUFY1 [31]	MKRN1 [19]			
				PRKAR1B [27]		TNIP1 [27]	ARMC10 [31]			
				MAPRE3 [27]		SNRNP70 [27]	AKAP9 [31]			
				STRN [27]						
				C2orf44 [32]						
				PPP1R21 [33]						
				SMEK2 [34]						

**Table:** (**b**)

Gene Fusions
ERAS-USP9X [16]
RSPO2-EIF3E [16]
RSPO3-PTPRK [16]
NCOA2-LACTB2 [15]
TCF7L2-VTI1A [15]
TCF7L2-RP11-57A14.3 [15]
SARAF (TMEM66)-NRG1 [35]
GTF3A-CDK8 [36]
NAGLU-IKZF3 [36]
RNF121-FOLR2 [36]
NFATC3-PLA2G15 [37]

**Table 2:** Approved drugs in solid tumors with known activity against recurrent mCRC gene fusions.

Gene Fusion	Drug	Disease	References
NTRK1,2,3-	Entrectinib	Solid tumors	[39,40,41]
	Larotrectinib	Solid tumors	[18]
ALK-	Crizotinib	NSCLC	[42]
	Entrectinib	NSCLC	[39,40,41]
	Alectinib	NSCLC	[43]
	Brigatinib	NSCLC	[44]
	Ceritinib	NSCLC	[45]
	Lorlatinib	NSCLC	[46]
ROS1-	Crizotinib	NSCLC	[47]
	Lorlatinib	NSCLC	[46]
	Entrectinib	NSCLC	[39,40,41]
RET-	Cabozantinib	RCC	[44]
	Vandetanib	Thyroid cancer	[48]
	Lenvatinib	RCC, Thyroid cancer	[49,50]
	Sorafenib	RCC, HCC	[51,52]
	Alectinib	NSCLC	[43]
	Regorafenib	CRC	[53]
	BLU-667 *	Solid tumors	[54,55]
	LOXO-292 ^+^	Solid tumors	[56]
FGFR2,3-	Erdafitinib	Urothelial carcinoma	[57]

* Not approved, planned FDA submission 2020. ^+^ FDA breakthrough therapy designation.
